# Web-based GTalign: bridging speed and accuracy in protein structure analysis

**DOI:** 10.1093/nar/gkaf398

**Published:** 2025-05-07

**Authors:** Justas Dapkūnas, Mindaugas Margelevičius

**Affiliations:** Institute of Biotechnology, Life Sciences Center, Vilnius University, Saulėtekio av. 7, 10257 Vilnius, Lithuania; Institute of Biotechnology, Life Sciences Center, Vilnius University, Saulėtekio av. 7, 10257 Vilnius, Lithuania

## Abstract

Accurate protein structure alignment is essential for understanding structural and functional relationships. Here, we introduce GTalign-web, a web-based implementation of GTalign, a spatial index-driven protein structure alignment tool, designed for accessibility and high-performance structural searches. Benchmarked against the DALI and Foldseek servers, GTalign-web demonstrates superior accuracy while maintaining rapid search times. Its utility is further highlighted in annotating uncharacterized proteins through searches against UniRef30. GTalign-web provides a useful resource for protein structure analysis and functional annotation and is available at https://bioinformatics.lt/comer/gtalign. This website is free and open to all users, and there is no login requirement.

## Graphical Abstract

**Graphical Abstract:**
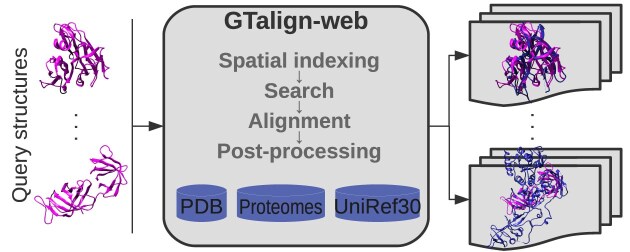

## Introduction

Understanding protein structure is fundamental to deciphering biological functions, inferring evolutionary relationships, and advancing drug discovery. Protein structure comparison and alignment play a crucial role in identifying structural similarities and enabling knowledge transfer between proteins. These methods also contribute to biomedical research by detecting conserved binding pockets and potential drug targets. Recent advances in protein structure prediction [1, 2] have dramatically increased the number of available structures [3, 4], creating new opportunities for structural analysis. However, this rapid growth underscores the need for efficient and accurate alignment tools to facilitate large-scale comparisons.

Despite its importance, protein structure alignment presents a significant computational challenge—finding optimal superposition, i.e. maximizing the number of aligned residues while minimizing the distance between them [5]. This process involves exploring a vast superposition space, making it computationally expensive. Achieving both speed and accuracy is particularly challenging. Existing tools, such as DALI [6], SSAP [7], CE [8], MAMMOTH [9], LGA [10], and TM-align [11], have made significant contributions but become computationally prohibitive when applied to large datasets. While some recent approaches [12–14] leverage pre-clustered structure databases to accelerate searches, computational performance limitations persist for very large datasets. Conversely, tools optimized for speed [15] often sacrifice sensitivity to biologically significant structural similarities, potentially overlooking evolutionary and functional relationships. This trade-off highlights the importance of protein structure alignment tools that achieve both efficiency and accuracy at scale, as demonstrated by the approach introduced in this work.

This article introduces GTalign-web, a web-based implementation of GTalign, a novel spatial index-driven protein structure alignment tool that combines high accuracy with high speed [16]. Designed for accessibility, GTalign-web provides a user-friendly platform for researchers across various disciplines, offering a valuable resource for protein structure analysis. The following sections describe its computational methodology, benchmark its performance, and illustrate its utility in biological applications. By providing a fast, accurate, and accessible platform for protein structure alignment, GTalign-web aims to facilitate the study of protein structure, function, and evolution, ultimately advancing biological research.

## Materials and methods

### New features of GTalign

GTalign-web, with its graphical and programmatic access, represents a new contribution. Several enhancements have been introduced to GTalign since its initial publication [16] to ensure the reliability and efficiency of GTalign-web services. First, the database caching mechanism has been improved, which is particularly important for handling large structure databases provided by GTalign-web. Second, support for the machine-readable JSON format has been implemented, facilitating data processing and allowing users to download standardized output.

### GTalign-web architecture

**Figure 1.**
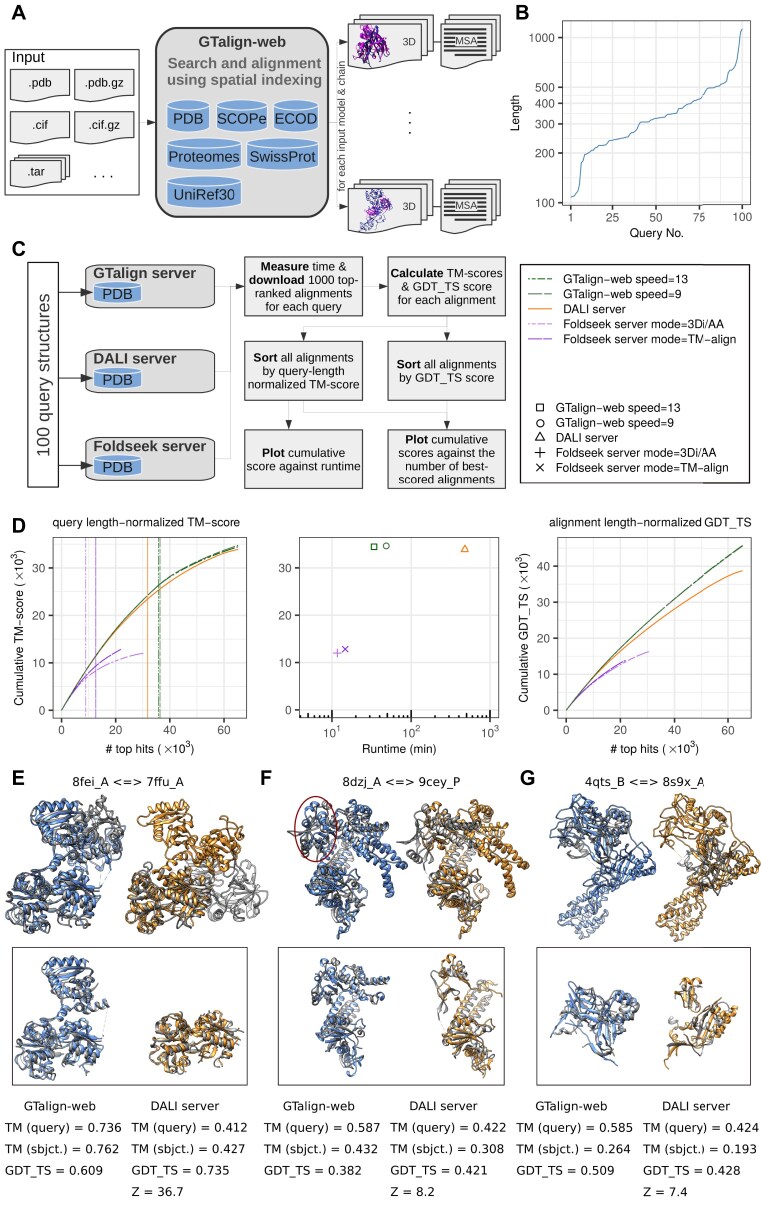
Overview of GTalign-web. (**A**) GTalign-web workflow. (**B**) Length distribution of query structures used in benchmarking. (**C**) Benchmarking setup. (**D**) Benchmark results. Vertical lines in the left panel indicate the number of alignments with a TM-score ≥0.5. The total number of top hits in the plots corresponds to the total obtained from the DALI server. For GTalign-web, the number of significant structural similarities is 97 576. Example alignments from benchmarking: structural alignments produced by GTalign-web and the DALI server for 8fei_A versus 7ffu_A (**E**), 8dzj_A versus 9cey_P (**F**), and 4qts_B versus 8s9x_A (**G**). Query structures are shown in gray, while subject structures are colored. Superpositions within frames highlight closely aligned regions (within 5 Å for GTalign-web or matched by DALI) to better visualize differences between methods. In panel (F), the spatial region corresponding to the domain not aligned by the DALI server remains visible due to rigid-body superposition. TM-scores normalized by query and subject (sbjct.) lengths are provided.

The GTalign-web workflow is illustrated in Fig. 1A. Designed for speed and accuracy, GTalign-web supports protein structure alignment for multiple query structures in various formats. Users can search against widely used and up-to-date structure databases spanning different levels of protein knowledge. For each query model and chain, GTalign-web returns pairwise structure alignments with database entries, which can be further analyzed. Users can visually inspect structural superpositions, select and generate multiple structure alignments (MSTAs), and download transformed database entries in bulk.

Jobs in GTalign-web are formatted as independent tasks and distributed across three GPUs, each handling one job at a time [17]. This setup enables parallel processing of three independent jobs, with each job processing all of its query structures simultaneously.

#### Input and output

GTalign-web accepts multiple input structures in PDB and PDBx/mmCIF formats, optionally gzipped. Users can also submit a TAR archive containing one or more structure files (Fig. 1A). The system automatically detects the input format, with a total file size limit of 10 MB.

The main results page provides links to individual result pages for each query model and chain. Each result page includes a summary and alignment section, displaying structure alignments, structural similarity scores, and other alignment statistics. Interactive visualization of structural superpositions is powered by NGL Viewer [18], while MSTAs (presented as multiple sequence alignments, MSAs) are rendered using MSAViewer [19].

#### Databases

GTalign-web supports searches across the following structure databases: RCSB PDB [20], SCOPe 40 [21], ECOD F70 [22], and proteins from 48 reference proteomes [3], UniProtKB/Swiss-Prot [23], and UniRef30 [24] (UniProtKB clustered at 30% sequence identity), with the last three datasets obtained from the AlphaFold Protein Structure Database [3]. UniRef30 was chosen over alternatives like UniRef50 to reduce large numbers of structurally redundant hits that arise at higher sequence identity thresholds.

#### Programmatic access via API

GTalign-web provides command-line and programmatic asynchronous access to running structure searches.

### Benchmarking

We benchmarked GTalign-web against two widely used protein structure alignment servers, DALI [13] and Foldseek [15], to evaluate its performance.

#### Queries

A set of 100 query structures was randomly selected by clustering [16] PDB protein chains associated with CRISPR–Cas systems at a TM-score [25] threshold of 0.4, then choosing singletons with the fewest hits in the PDB. The selected queries were distributed by length as follows: 10% between 100 and 200 residues, 30% between 200 and 300 residues, 30% between 300 and 400 residues, and 30% longer than 400 residues (Fig. 1B). Not all query structures are directly related to CRISPR–Cas, as the initial dataset was retrieved using a keyword-based search. For consistency in interpretation, HETATM records and residues lacking at least one of the N, CA, C, or O atoms were removed from the query structures.

#### Setup

The three web servers were queried with the same 100 structures to search the PDB (Fig. 1C). Once all jobs were completed, runtimes were recorded. For each query, the top 1000 alignments from each server were downloaded. TM-scores and GDT_TS [10] scores were then calculated to evaluate alignment accuracy. The alignments were sorted by TM-score and GDT_TS, and the accuracy rates for each server were visualized in comparative plots.

#### Evaluation metrics

The DALI and Foldseek servers were queried via the command line, with all queries submitted at once for DALI and in multiple permitted quotas for Foldseek. GTalign-web was queried with three concurrent submissions. Measured runtimes exclude inter-submission delays and queue wait times.

Alignment accuracy was first evaluated by the TM-score [25], a global structural similarity measure where 1 indicates a perfect match. TM-align [11] was used to calculate TM-scores, constrained by the given alignment [16]. TM-scores were normalized by query length, reducing alignment significance for proteins much smaller than the query. Since DALI and Foldseek assess significance using unnormalized scores and are sensitive to larger length ratios, the query length-normalized TM-score provides a fairer mutual comparison.

Additionally, alignment accuracy was evaluated using GDT_TS [10], an independent measure. A modified TM-align [16] was used to normalize GDT_TS scores by the number of aligned residues. Accuracy evaluations within alignment boundaries tend to favor local alignment methods like Foldseek, as they do not penalize unaligned regions.

## Results and discussion

### Benchmark results

The benchmark results are shown in Fig. 1D. It is important to note that this evaluation is not strictly objective due to variations in PDB maintenance. For instance, GTalign-web allows searches across all models, whereas DALI considers only the first structure models. To ensure a fair comparison, GTalign-web results were filtered to include only single models. However, the Foldseek server’s PDB100 appears to retain a single representative structure at 100% sequence identity and was last updated on 1 January 2024 at the time of writing. Despite these differences, previous rigorous evaluations [16] of these and other methods suggest that this benchmark reasonably reflects the relative performance of the web servers.

#### Runtime and alignment accuracy

Figure 1D presents the results of GTalign-web using two speed settings (default = 13) optimized for best efficiency. (Lowering the speed setting increases sensitivity to structural similarity.) GTalign-web achieved the highest rate of accurate alignments among the benchmarked servers, as assessed by global (left panel) and local structural similarity (right panel). It processed all queries in 34 min (48 min at speed = 9), significantly faster than DALI (477 min) and reasonably slower than Foldseek, which took 12 min (15 min in TM-align mode) (middle panel).

#### Examples

This subsection presents several example alignments produced by GTalign-web and the DALI server. The first example compares conalbumin (8fei_A) and human serum transferrin (7ffu_A), both members of the transferrin family (Fig. 1E). These proteins share 38% sequence identity, making them relatively straightforward even for sequence alignment methods. Structurally, 8fei_A consists of two domains, each containing swapped subdomains. While GTalign-web captures the overall global structural similarity with a high TM-score of 0.736, DALI aligns only one domain, resulting in a significantly lower TM-score of 0.412. However, DALI’s GDT_TS score is higher (0.735) because its alignment excludes a slightly differently positioned domain, which GTalign-web still aligns.

Another example compares a subunit from an RNA-guided DNA endonuclease of a Cas12f complex (8dzj_A) with a eukaryotic RNA-guided endonuclease, Fanzor (9cey_P) (Fig. 1F). Despite sharing the same overall architecture and fold, these two-domain proteins have only 4% sequence identity. Their differing domain orientations and divergent regions cause DALI to align only one domain accurately, leaving the other unaligned. In contrast, GTalign-web aligns both domains (with the domain unaligned by DALI highlighted in the figure), resulting in a higher global similarity (TM-score = 0.587) but lower local similarity (GDT_TS = 0.382) due to regional differences introduced by the additionally aligned domain.

The final example compares a Cas protein (Csm4) from the type III-A CRISPR–Cas system (4qts_B) with the Cas7-5-11 subunit of the type III-Dv CRISPR–Cas complex (8s9x_A) (Fig. 1G). The query (4qts_B) is a single-domain protein that shares 8% sequence identity with 8s9x_A. GTalign-web and DALI align 4qts_B to different domains of 8s9x_A, with GTalign-web producing a more accurate alignment both globally (TM-score = 0.585) and locally (GDT_TS = 0.509).

### Case studies of distant relationships from UniRef30

To showcase GTalign-web’s utility in protein annotation, we selected two chains with the fewest hits in the PDB and searched them against UniRef30, completing the searches in 28 min. This subsection presents several case studies based on the results.

**Figure 2.**
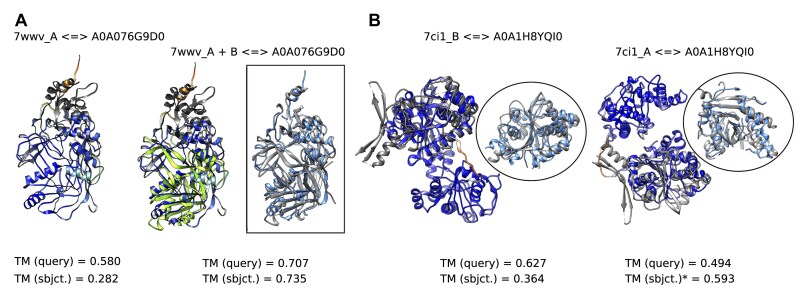
Case studies from UniRef30 searches. (**A**) Superposition of A0A076G9D0 with 7wwv_A (left) and with chains A and B of 7wwv (right), where 7wwv_B is highlighted in a light hue. (**B**) Superposition of 7ci1_B with the second domain of A0A1H8YQI0 (left) and of 7ci1_A with its first domain (right). In all panels, query structures are shown in gray, while subject structures are colored by model confidence, ranging from orange (very low: pLDDT<50) to blue (very high: pLDDT>90). Superpositions within frames emphasize closely aligned regions (within 5 Å), showing 7wwv chains A and B (gray) aligned to A0A076G9D0 (colored) in panel (A), and 7ci1 chains B and A (gray) aligned to the corresponding domains of A0A1H8YQI0 (colored) in panel (B). The asterisk denotes the TM-score calculated for the first domain of A0A1H8YQI0. Images were prepared using UCSF Chimera [26].

The first protein analyzed is the Csy1 subunit of a type I-F CRISPR–Cas complex (7wwv_A). Among the 63 hits with a query length-normalized TM-score >0.3, 26 were CRISPR-associated proteins, 2 were nucleases, 27 were uncharacterized proteins, and 7 lacked command line-retrieved descriptions. These results demonstrate high reliability, as all annotated proteins except one were nucleases. We further examined the least significant hit, an uncharacterized protein (A0A076G9D0) with 4% sequence identity to the query. Despite significant structural similarity (Fig. 2A), its functional inference remains limited due to partial structural coverage [TM(sbjct.) = 0.282]. However, when the Csy2 subunit (7wwv_B) was included, aligning both subunits with A0A076G9D0 revealed a strong global structural match [TM(sbjct.) = 0.735]. Since the Csy1 subunit is involved in recognizing a protospacer adjacent motif (PAM) in DNA [27], these findings suggest A0A076G9D0 may function as part of a CRISPR–Cas complex or as a DNA-binding protein.

The second protein analyzed is a chain of the anti-CRISPR homodimer protein AcrVA2 (7ci1_B), which binds to Cas12a to inhibit CRISPR–Cas immune function [28]. Among the 342 hits with a query length-normalized TM-score >0.3, all 273 proteins with retrieved descriptions were uncharacterized. Here, we examine one of these hits, A0A1H8YQI0, which shares 8% sequence identity with the query and exhibits partial coverage. A0A1H8YQI0 is a two-domain protein, with its second domain (residues 274–543) sharing significant structural similarity with the query (Fig. 2B). Notably, the first chain of AcrVA2 (7ci1_A) closely matches the first domain (residues 1–238) of A0A1H8YQI0. The structural model of A0A1H8YQI0 is predicted with high confidence, except for the linker region between its domains, which makes their relative orientation uncertain. Therefore, this result provides insight into both the domain arrangement of A0A1H8YQI0 and its potential inhibitory function.

### Comparison with existing tools

GTalign-web enables rapid searches across various structural databases, with UniRef30 being particularly noteworthy. GTalign-web can search UniRef30—currently comprising over 25 million structures—within minutes. To the best of our knowledge, among existing tools, only the Foldseek server offers searches at this scale or larger. However, Foldseek is deliberately optimized for speed, often at the cost of accuracy and sensitivity to structural similarity, as demonstrated in the previous study [16] and this work. Consequently, many significant structural relationships often remain undetected.

In contrast, long runtimes of DALI prevent its server from supporting searches against very large structure databases, even with preprocessing [12, 13]. While the DALI server allows searches across the reference proteomes from the AlphaFold database, this dataset contains only 564 329 structures. For comparison, the PDB used for benchmarking GTalign-web included over 1 million models and chains.

Given these considerations, we believe GTalign-web fills an important gap in the current landscape of protein structure comparison tools and web servers by offering both fast and accurate alignments.

## Conclusion

GTalign-web, a newly developed web server, integrates the robust computational framework of GTalign [16] with an intuitive interface to facilitate access to high-performance protein structure alignment. GTalign-web addresses key limitations of existing web servers by delivering both speed and sensitivity to significant structural similarities, even for large-scale datasets. Its ability to efficiently search extensive structure databases, such as UniRef30, provides valuable insights into protein annotation and functional characterization. With GTalign-web, we aim to bridge the gap between computational innovation and practical usability.

## Data Availability

The source code for GTalign-web’s backend and frontend is available at https://github.com/minmarg/gtalign-web-backend (https://doi.org/10.5281/zenodo.15276061) and https://github.com/chemikeris/comer_web (https://doi.org/10.5281/zenodo.15277924). Query structures, data, permanent links to GTalign-web’s benchmark and case study results, and scripts for benchmarking and producing graphs are accessible at https://github.com/minmarg/gtalign-web-benchmark (https://doi.org/10.5281/zenodo.15276216). All structure databases used within the GTalign-web framework are open-access resources, with availability details provided in their respective references.
